# The association between time-of-day of habitual exercise training and changes in relevant cancer health outcomes among cancer survivors

**DOI:** 10.1371/journal.pone.0258135

**Published:** 2021-10-12

**Authors:** Adriana M. Coletta, Mary C. Playdon, Kelly G. Baron, Mei Wei, Kristen Kelley, Christos Vaklavas, Anna Beck, Saundra S. Buys, Jonathan Chipman, Cornelia M. Ulrich, Darren Walker, Shelley White, Sonal Oza, Rebecca W. Zingg, Pamela A. Hansen

**Affiliations:** 1 Department of Health & Kinesiology, The University of Utah, Salt Lake City, UT, United States of America; 2 Huntsman Cancer Institute at the University of Utah, Salt Lake City, UT, United States of America; 3 Department of Nutrition and Integrative Physiology, The University of Utah, Salt Lake City, UT, United States of America; 4 Department of Family and Preventive Medicine, The University of Utah, Salt Lake City, UT, United States of America; 5 Department of Internal Medicine, The University of Utah, Salt Lake City, UT, United States of America; 6 Department of Population Health Sciences, The University of Utah, Salt Lake City, UT, United States of America; 7 Division of Physical Medicine and Rehabilitation, The University of Utah, Salt Lake City, UT, United States of America; Universidade Federal de Mato Grosso do Sul, BRAZIL

## Abstract

**Objective:**

To assess the relationship between time-of-day of exercise training and changes in relevant cancer health outcomes among cancer survivors.

**Methods:**

Retrospective analysis of data collected from 2016–2019 from a hospital-based exercise oncology program. Descriptive statistics were calculated for demographic, clinical, and exercise timing characteristics (e.g. AM, PM, or mix) among survivors with available data for exercise training time (n = 233). For the total sample and a breast cancer sub-analysis, univariate analysis of covariance, adjusted for age, was carried out by exercise training time, for change in the following outcomes collected during the program’s assessment sessions: cardiorespiratory fitness and muscular endurance (human performance variables), physical function, anthropometrics, self-reported fatigue, and quality of life (QoL). Change in body mass index (BMI) and body weight was included in the breast cancer analysis.

**Results:**

Overall, 37.3% of survivors habitually engaged in AM exercise (e.g. ≥ 75% AM training), 34.3% in PM exercise, and 28.3% in a mix of AM and PM exercise training throughout the program. Median time in the program was 17 weeks. Significant improvements in most human performance and physical function variables were observed in the total sample regardless of exercise training time-of-day. Among breast cancer survivors, PM but not AM or mixed was associated with improvements in fitness, and lower-body muscular endurance and function. Mixed exercise timing was linked with greater increase in waist circumference (total sample: 3.02cm, 95%CI 1.55, 4.49; breast cancer: 3.57cm 95%CI 0.96, 6.18), body weight (breast cancer: 1.6kg, 95%CI 0.3, 2.8) and BMI (breast cancer: 0.6kg/m^2^, 95%CI 0.1, 1.0). AM and PM exercise, but not mixed, was associated with improvements in fatigue and QoL.

**Conclusion:**

Time-of-day of exercise training may differentially impact changes in human performance and physical function variables. Mixed exercise training time may result in less favorable outcomes related of weight management variables among cancer survivors.

## Introduction

Substantial evidence supports the relationship between exercise engagement after a cancer diagnosis and attenuation of cancer treatment related side effects, improvements in quality of life, and reduction in risk of cancer-specific and all-cause mortality [1–3]. Specific components of exercise prescription, such as type of exercise (e.g. aerobic exercise or strength training), total weekly duration of aerobic exercise (e.g. minutes per week), and exercise intensity (e.g. high-intensity interval training), can be manipulated to optimize changes in relevant cancer health outcomes [1, 4–7]. For example, 30-minutes of moderate-intensity aerobic exercise three times per week is significantly associated with reductions in cancer-related fatigue among cancer survivors of varying cancer types [1]. Additionally, evidence suggests that high-intensity interval training may be superior compared with moderate-intensity continuous aerobic exercise for improving cardiorespiratory fitness in colorectal cancer survivors [7]. This growing body of evidence supports the utility of exercise prescription and programming to promote favorable changes in relevant cancer health outcomes among cancer survivors.

In other clinical populations, such as individuals with metabolic disease (e.g. obesity, type 2 diabetes), in addition to manipulation of exercise prescription, there is evidence to suggest time-of-day of exercise engagement may affect disease related health outcomes, such as weight status and body composition, glycemic control, and physical function. These health outcomes are also relevant in the context of cancer survivorship, such that an obesity weight status, adverse body composition (e.g. higher fat mass, lower lean mass), poor glycemic control, and declines in physical function/human performance are not only common cancer treatment-related side effects [8–12], but are also linked with poor survival across varying cancer types [13–20]. In other clinical populations, for example individuals with obesity, habitually engaging in moderate to high-intensity continuous aerobic exercise in the morning hours (AM) has been shown to be more effective in promoting weight loss, and reductions in body fat and waist circumference compared with habitually exercising in the afternoon and evening hours (PM) [21, 22]. Additionally, among individuals with type 2 diabetes but without cancer, habitually engaging in aerobic and resistance exercise in the morning hours has demonstrated superior utility in improving glycemic control [23] compared with habitual engagement in afternoon and evening exercise. On the other hand, afternoon and evening exercise is associated with greater exercise capacity, which may contribute to observations related to optimal changes in markers of human performance and physical function with habitual engagement in afternoon and evening exercise [24]; but mechanisms have yet to be elucidated. Collectively, the evidence suggests that time-of-day of exercise engagement across various non-cancer populations may differentially impact changes in a range of health outcomes that are also relevant to cancer survivors.

The fact that time-of-day of exercise engagement influences relevant cancer outcomes in other clinical populations provides a rationale for manipulating exercise training time based on outcomes for cancer survivors. Identifying optimal timing of exercise may therefore augment current best practices for exercise prescription across the cancer care continuum. To our knowledge, missing in the literature is investigation of this concept in cancer survivors. Therefore, the purpose of the present study was to assess the relationship between habitual time-of-day of exercise training and changes in relevant cancer health outcomes among cancer survivors of varying cancer types and stages participating in a hospital-based exercise oncology program. Consistent with available evidence [21, 22, 24], we hypothesize that habitual AM exercise training, compared with PM, would be associated with greater improvements in weight management variables (e.g. body weight, waist circumference, and body mass index), and habitual PM training, compared with AM, would be associated with greater improvements in human performance and physical function variables (e.g. cardiorespiratory fitness, muscular strength and endurance, lower body and whole body physical function) among cancer survivors.

## Materials and methods

### Study design, participants, and exercise oncology program

This is a retrospective analysis of data collected from 2016–2019 from the Huntsman Cancer Institute’s (HCI) on-site exercise oncology program, the Personal Optimism With Exercise Recovery program (POWER). The protocol and waiver of informed consent was approved by the University of Utah Institutional Review Board. The patient population and exercise oncology program are described in detail elsewhere [25]. Briefly, POWER is an on-site exercise oncology program offered to all patients treated at HCI. POWER includes a physical and medical assessment carried out by a team of physical medicine and rehabilitation physicians and exercise physiologists. Participants in POWER receive an individualized exercise prescription based on the physical and medical assessment they complete at the start of the program. The exercise prescription includes goals for both aerobic and resistance exercise training. Exercise training delivery (e.g. supervised, home-based via telehealth; supervised, in-person at the wellness center gym; unsupervised, home-based) and time-of-day of exercise training (e.g. morning or afternoon/evening) and assessments is dictated by patient preference. Once engaged in the program, participants may choose to complete a follow-up assessment to monitor progress, which mirrors the initial assessment. If a follow-up assessment is completed, the exercise prescription is then altered based on these results. At this point, participants may still choose to continue the program supervised in-person, supervised home-based via telehealth, or they may choose to transition to unsupervised home-based. The average length of time among patients who participated in the program and completed at least one follow-up assessment to review progress was 25 weeks [25]. Our study sample consists of patients with a history of any invasive cancer.

### Health outcomes evaluated at program assessments

Procedures related to the health outcomes evaluated at the initial assessment and follow-up assessment are outlined in detail elsewhere [25]. Sample sizes for each outcome vary because not all survivors were able to complete all tests at their assessment (initial or follow-up) due to limitations from treatment. These outcomes include: cardiorespiratory fitness measured by relative peak aerobic capacity (peak VO2) and peak metabolic equivalents (METs), estimated using a modified Bruce protocol and American College of Sports Medicine metabolic calculations for estimating energy expenditure; whole body muscular endurance measured by ten repetition maximum (10 RM) testing for chest press, latissimus pulldown, and leg press; physical function and strength measured by the timed up and go test, 30-second chair-stand test, and handgrip strength test; anthropometrics including waist and hip circumference measurements; and fatigue and quality of life measured with the Functional Assessment of Chronic Illness Therapy- Fatigue (FACIT-Fatigue) version 4 and Functional Assessment of Cancer Therapy- General (FACT-G7) questionnaires respectively. Cardiorespiratory fitness, muscular strength and endurance, and physical function variables are considered markers of human performance and physical function.

### Data extraction for exercise timing

Date and time-of-day of exercise training sessions were extracted from the electronic medical record by one of our investigators, DW, via the University of Utah’s electronic data warehouse. Medical record numbers of patients who participated in the POWER program between 2016 and 2019 and completed a follow-up assessment to monitor progress were provided (n = 239), and data were extracted for these specific cases. For each participant, the date of the initial and follow-up POWER assessment was matched with the dates of the exercise training sessions by another one of our investigators, AMC. Exercise training data were available for 233 of the 239 medical records queried. Authors agreed upon the following criteria to classify individuals as habitual AM or PM exercisers: patients who completed at least 75% of their exercise training sessions before noon were considered individuals who completed exercise training in the morning (AM), and those who completed at least 75% of training sessions after noon were considered afternoon/evening (PM) exercisers. The remaining participants were considered individuals who exercised in a mixed routine of morning and afternoon exercise training. The POWER program offers exercise training from 7:00am to 7:00pm Monday through Friday. AM assessments included a start time between 8:00am and 11:59am. PM assessments included a start time between noon and 3:00pm.

### Statistical methods

Descriptive statistics were calculated for demographic and clinical characteristics at the initial assessment among individuals with data for time-of-day of exercise training (n = 233). Frequencies for time-of-day of follow-up assessment among participants who habitually engaged in AM or PM exercise was compared separately to determine if the time-of-day of the follow-up assessment was consistent with time-of-day of exercise training (e.g. the proportion of participants with habitual AM exercise training and an AM follow-up assessment). Change variables were computed as the difference between the follow-up assessment value and the initial assessment value. Univariate analysis of covariance across habitual exercise training time (AM, PM, mix), adjusted for age at initial assessment, was carried out for the change variables in the total sample. We also conducted a sub-analysis among breast cancer survivors, which was the predominant cancer type. Change in body mass index (BMI) and body weight, controlled for age, was included in the breast cancer analysis, since weight gain is a common treatment related side effect in this patient population, and weight gain during treatment is associated with poor survival outcomes [26]. We did not include body weight or BMI assessment in the analysis for the total sample due to the heterogeneity in cancer types included and the notion that change in body weight may be harmful or beneficial pending on cancer type; for example, weight gain among breast cancer survivors is associated with poor prognosis, yet weight loss in pancreatic cancer is associated with poor prognosis. We also compared our findings with established cut-offs for minimal clinically important difference (MCID) for peak VO2 (+1.5–2.0 ml/kg/min) and METs (+1.0 MET) [27, 28], handgrip strength (+5–6.5 kg) [29], 10RM leg press (+5.6 kg) [30], timed up and go test (1–1.5 seconds) [31–34], 30-second chair stand test (≥ 2 seconds) [33], FACIT-Fatigue (+3 units to total score) and FACT-G7 (≥ 4 units to total score) questionnaires [35, 36]. We did not carry out a sub-analysis by exercise training delivery (e.g. supervised, home-based via telehealth; supervised, in-person at the wellness center gym; unsupervised, home-based) because we previously reported that only two patients from this dataset elected a hybrid of in-person and home-based training, while the rest completed their training supervised, in-person at the Wellness Center gym [25]. Assessment data are presented as means and 95% confidence intervals (CI). All data were analyzed with SPSS statistical software package, version 26 (Chicago, IL).

## Results

Characteristics of all participants who completed a follow up assessment are presented elsewhere [25]. Among patients with data related to time-of-day of exercise training (n = 233), 72% were female, 94% were white, and 95% were non-Hispanic. The majority of these cancer patients had breast cancer (40.8%), followed by prostate cancer (10.7%) and endometrial cancer (6.4%), and most patients had cancer stage I-III (68.7%) compared with stage IV (14.2%), no stage (12.9%), as in patients with hematological malignancies, or an unknown stage (4.3%). The average age was 61.6±13.2 years, and the average BMI was 28.3±6.5 kg/m^2^ (n = 231). BMI was missing for two of the participants in the sample. Regarding time-of-day of exercise training sessions in the POWER program, 37.3% (n = 87) habitually exercised in the morning, whereas 34.3% (n = 80) habitually exercised in the afternoon/evening, and 28.3% (n = 66) engaged in a mix of morning and afternoon/evening exercise training time. Among AM exercisers, the majority, 73%, completed their follow-up assessment in the morning hours. Among PM exercisers, most, 66%, completed their follow-up assessment in the afternoon/evening hours. Similar findings regarding time-of-day of exercise training and follow-up assessment were observed among breast cancer survivors. Among all cancer survivors, the average number of training sessions between initial and follow-up assessment was 13 for AM exercisers, 16 for PM exercisers, and 18 for those engaged in a mix of training time. Median and interquartile range for time between initial and follow-up assessment for the entire sample was 17 weeks (Q1: 14 weeks, Q3: 28 weeks)), and by exercise training time: 16 weeks (Q1: 13 weeks, Q3: 25 weeks) for AM, 16 weeks (Q1: 14 weeks, Q3: 25 weeks) for PM exercisers, and 20 weeks (Q1: 14 weeks, Q3: 37 weeks) for mixed exercise training time.

**Fig 1:**
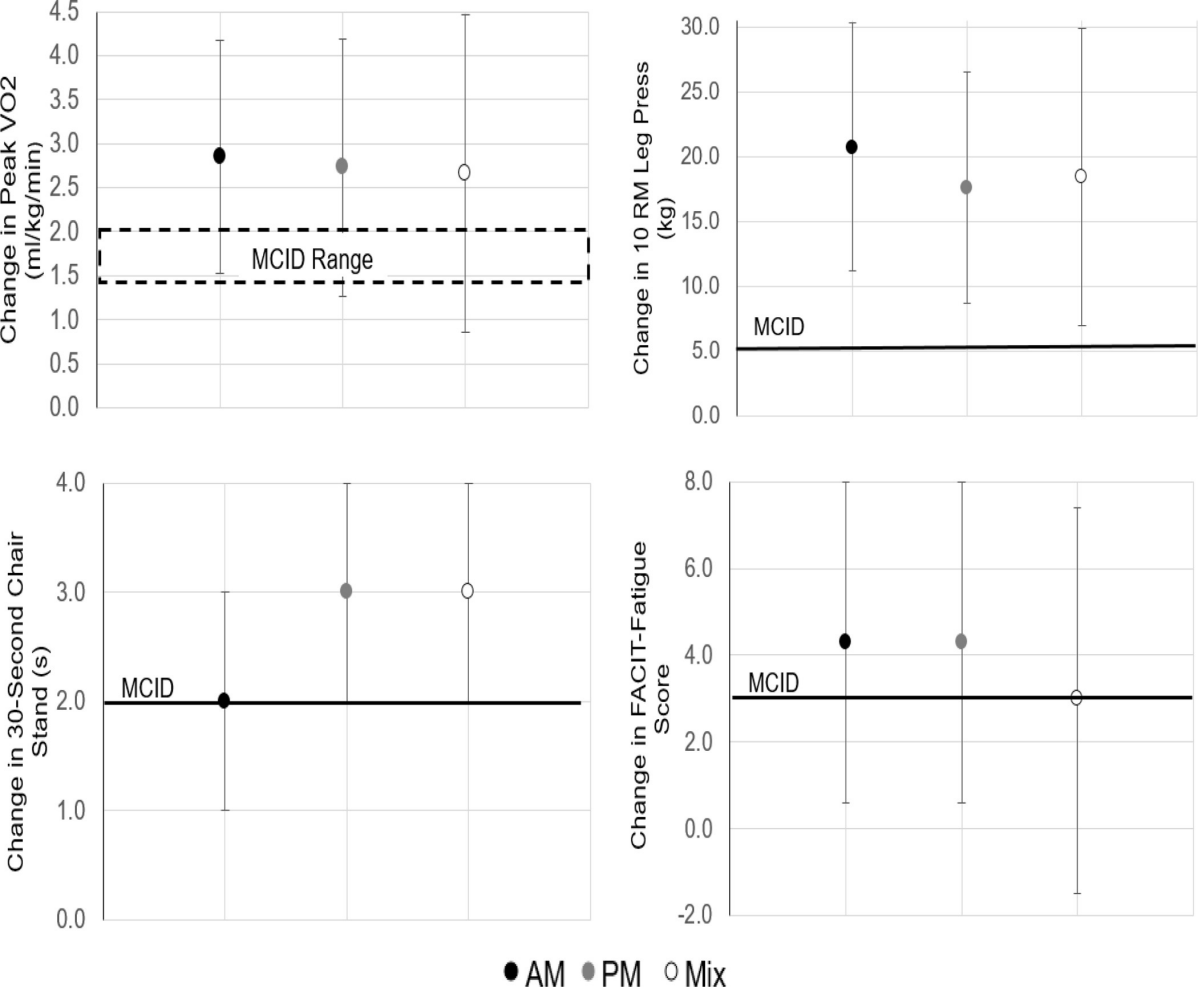
Statistically significant changes in health outcomes that exceeded the minimal clinically important difference (MCID) threshold for the total sample. Data presented as mean and 95% confidence intervals.

Table 1 presents the mean change in relevant cancer health outcomes by time-of-day of habitual exercise training for the total sample and among breast cancer survivors. In the total sample, patients experienced significant improvements in cardiorespiratory fitness variables (peak VO2 and METs), lower body physical function (30-second chair stand test), and lower and upper body muscular endurance (10 RM leg press, latissumus pulldown, and chest press), regardless of time-of-day of exercise training. Changes in peak VO2, 10 RM leg press, and 30-second chair stand test exceeded the threshold for MCIDs for all training times (AM, PM, mix). Self-reported quality of life (FACT-G7) was only significantly improved among individuals who habitually exercised in the afternoon/evening. The change in FACT-G7 score did not exceed the threshold for MCID. Self-reported fatigue was significantly improved among both AM and PM exercisers, and change in these scores exceeded MCID, but this was not observed among those who engaged in a mix of exercise training time. PM exercise training time or a mix resulted in significant improvements in whole-body physical function (timed up and go); but this change did not exceed the threshold for MCID. Furthermore, engaging in a mix of exercise training time was associated with a significant increase in waist circumference. No other significant changes within groups were observed. Fig 1 compares change in outcomes to available MCID thresholds for the total sample.

**Table 1:** Mean change in health outcomes by time-of-day of habitual exercise training.

Variable	AM			PM			Mix		
	n	Mean	95%CI	n	Mean	95%CI	N	Mean	95%CI
**Total Sample (n = 233)**									
Peak VO2 (mL/kg/min)	59	2.85	(1.53, 4.17)	48	2.73	(1.26, 4.19)	31	2.66	(0.86, 4.46)
Peak METs	59	0.81	(0.44, 1.19)	48	0.78	(0.36, 1.20)	31	0.76	(0.25, 1.28)
10 RM Chest Press (kg)	55	6.1	(3.6, 8.5)	61	5.2	(2.9, 7.5)	31	3.8	(0.6, 7.1)
10 RM Leg Press (kg)	48	20.7	(11.2, 30.3)	55	17.6	(8.7, 26.5)	33	18.4	(7.0,29.9)
10 RM Latissumus Pull Down (kg)	56	7.7	(5.0, 10.3)	60	6.4	(3.8, 9.0)	34	7.8	(4.4, 11.2)
Timed Up and Go (seconds)	80	-0.13	(-0.53, 0.27)	75	-0.43	(-0.84, -0.01)	58	-0.61	(-1.22, -0.09)
30-second Chair Stand (repetitions)	84	2	(1, 3)	78	3	(2, 4)	61	3	(2, 4)
Right Hand Grip (kg)	84	0.9	(-0.2, 2.0)	78	0.9	(-0.3, 2.0)	65	0.4	(-0.8, 1.7)
Left Hand Grip (kg)	84	0.1	(-1.1, 1.3)	79	0.5	(-0.8, 1.7)	65	0.3	(-1.1, 1.6)
Waist Circumference (cm)	78	-0.58^a^	(-1.89, 0.72)	78	-0.25^a^	(-1.55, 1.05)	61	3.02	(1.55, 4.49)
Hip Circumference (cm)	79	0.17	(-1.60, 1.95)	78	-0.59	(-2.38, 1.19)	62	-1.39	(-3.39, 0.61)
FACIT-Fatigue Score	57	4.3	(0.6, 8.0)	57	4.3	(0.6, 8.0)	39	3	(-1.5, 7.4)
FACT-G7 Score	54	1.5	(0.1, 3.0)	61	2.0	(0.7, 3.4)	38	1.4	(-0.3, 3.2)
**Breast Cancer Survivors (n = 95)**									
Peak VO2 (mL/kg/min)	28	2.14	(-0.03, 4.31)	24	2.51	(0.15, 4.87)	8	3.33	(-0.69, 7.36)
Peak METs	28	0.61	(-0.01, 1.23)	24	0.72	(0.04, 1.39)	8	0.95	(-0.20, 2.10)
10 RM Chest Press (kg)	26	6.5	(2.7, 10.4)	25	4	(0.1, 7.9)	12	0.4	(-5.2, 6.1)
10 RM Leg Press (kg)	23	20.2	(7.4, 32.9)	23	25.0	(12.3, 37.7)	14	21.5	(5.2, 37.9)
10 RM Latissumus Pull Down (kg)	27	8.5	(4.0, 12.9)	23	5.7	(0.8, 10.5)	14	6.5	(0.3, 12.7)
Timed Up and Go (seconds)	36	0.37^a^	(-0.71, -0.03)	30	-0.45	(-0.82, -0.07)	23	-0.97	(-1.39, -0.54)
30-second Chair Stand (repetitions)	36	2	(0, 4)	31	3	(1, 5)	24	4	(2, 6)
Right Hand Grip (kg)	36	0.9	(-1.0, 2.7)	31	0.2	(-1.8, 2.2)	26	0.3	(-1.8, 2.5)
Left Hand Grip (kg)	36	0.1	(-1.7, 2.0)	31	-0.6	(-2.6, 1.4)	26	1.1	(-1.1, 3.3)
Waist Circumference (cm)	35	-1.06^a^	(-3.26, 1.15)	31	-1.30^a^	(-3.65, 1.04)	25	3.57	(0.96, 6.18)
Hip Circumference (cm)	35	0.92	(-2.87, 4.71)	31	-1.41	(-5.43, 2.61)	25	-4.20	(-8.68, 0.28)
Weight (kg)	37	0.7^b^	(-0.3, 1.8)	31	-1.0	(-2.1, 0.1)	25	1.6^b^	(0.1, 1.0)
BMI (kg/m^2^)	37	0.3^b^	(-0.1, 0.6)	31	0.4	(-0.8, 0.02)	25	0.6^b^	(0.1, 1.0)
FACIT-Fatigue Score	25	5	(-1.5, 11.5)	20	5.4	(-1.9, 12.7)	18	2.9	(-4.7, 10.5)
FACT-G7 Score	24	2.4	(-0.1, 4.8)	23	2.4	(-0.1, 4.9)	16	1.9	(-1.1, 4.8)

Results adjusted for age at initial assessment. Shaded cells indicate statistically significant (p<0.05) findings.

^a^Significantly different from mix (p<0.05) in pairwise comparison.

^b^Significantly different from PM (p<0.05) in pairwise comparison. Peak VO2 = peak oxygen consumption, METs = Metabolic Equivalents, 10RM = 10 Repetition Maximum, FACIT = Functional Assessment of Chronic Illness Therapy, FACT = Functional Assessment of Cancer Therapy.

**Fig 2:**
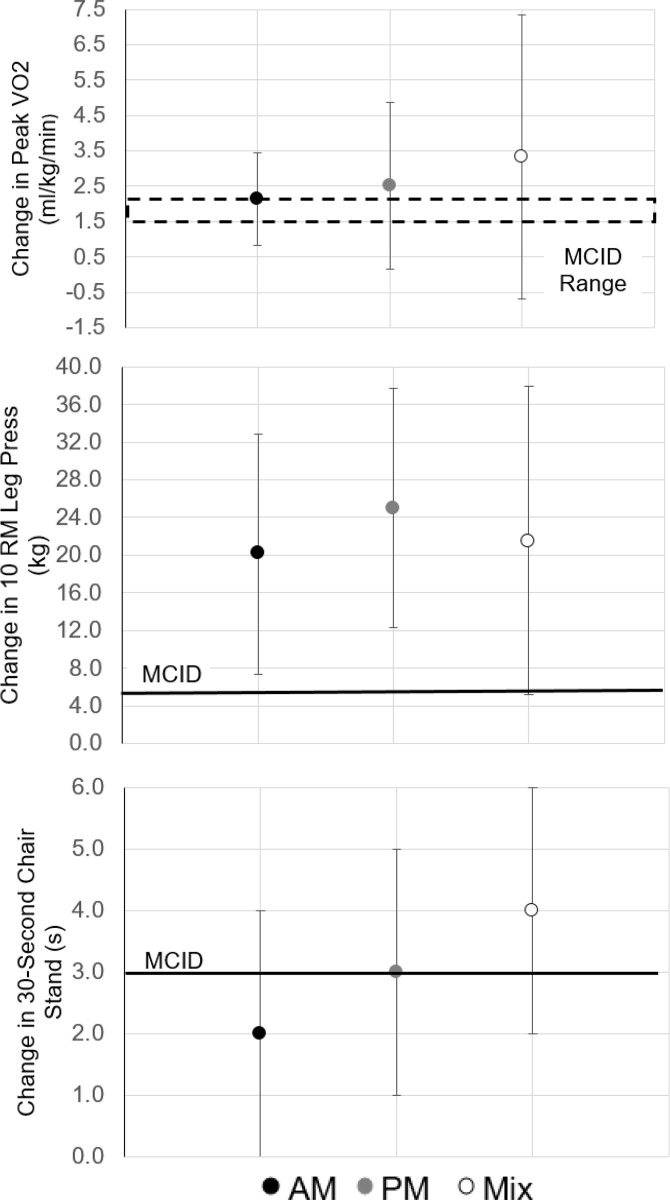
Statisticially significant changes in health outcomes that exceeded the minimal clinically important difference (MCID) threshold among breast cancer survivors. Data presented as mean and 95% confidence intervals.

Among breast cancer survivors, significant improvements were observed in whole-body physical function (timed up and go), lower body muscular endurance (10RM leg press), and one measure of upper body muscular strength (10 RM latissumus pulldown) regardless of the time-of-day of exercise training. Only changes in 10 RM leg press exceeded the threshold for MCID. Significant improvements in cardiorespiratory fitness variables were only observed among PM exercisers, and the change in peak VO2 exceeded the threshold for MCID. Lower body physical function was significantly improved in breast cancer survivors who engaged in PM exercise or a mix throughout their training program, with changes exceeding MCID. However upper body muscular endurance was only significantly improved in AM and PM exercisers but not in a mix. Further, breast cancer survivors who engaged in a mix of exercise training time throughout their program experienced significant increases in waist circumference, body weight, and BMI. No other significant changes within groups were observed. Fig 2 compares change in outcomes to available MCID thresholds for the total sample.

## Discussion

This study assessed the relationship between time-of-day of habitual exercise training and changes in relevant cancer health outcomes among cancer survivors who participated in a hospital-based exercise oncology program. Among the total sample, a statistically significant relationship was not observed between a specific time-of-day of exercise training and improving the majority of markers assessed for human performance and physical function (e.g., cardiorespiratory fitness variables, upper and lower body muscular endurance, and lower body physical function). Among breast cancer survivors, we observed significant associations specifically between PM exercise and the following human performance and physical function variables: cardiorespiratory fitness variables, lower body muscular endurance, and lower body physical function. There were still some variables where significant improvements were observed for both AM and PM exercise training (one measure of upper body muscular endurance- 10 RM chest press), and in some cases, even a mix of exercise training time (one measure of upper body muscular endurance measured by 10 RM latissumus pull down, and whole body physical function measured by timed up and go test). Our findings related to MCIDs support the utility of our hospital-based exercise oncology program to improve health outcomes associated with quality of life and survival among cancer survivors.

A possible explanation for our findings related to time-of-day of exercise training may be due to our observation that there was consistency between both time-of-day of exercise training and time-of-day of follow-up assessment time. For example, the majority of survivors who habitually exercised in the morning also completed their follow-up assessment in the morning. Previous research suggests that consistency with time-of-day of exercise training will result in the most significant improvements in human performance variables [18]. For example, a competitive marathon runner will exhibit the best performance when his/her exercise training sessions occur at the same time-of-day as the marathon. Circadian biology may underpin this observation, including diurnal variations in body temperature, substrate metabolism, neuromuscular function, and hormones [24, 37]. Consistent exercise timing may serve as an external cue to attenuate circadian rhythm disruption by regulating circadian gene expression and other biological mechanisms [18]. Disruptions in diurnal rhythms can negatively impact human performance [37]. Unfortunately, biomarkers of circadian rhythm disruption, such as sleep quality, melatonin levels, and amplitude of rhythms measured by actigraphy, were unavailable for the current analysis; however, declines in sleep quality and circadian rhythm disruption are common side effects of cancer treatment [38–40]. Furthermore, a mix of exercise training time may reflect other instabilities in an individual’s schedule, such as inconsistent sleep/wake cycle, meal timing, work schedule, availability to get an appointment relative to other cancer treatment appointments.

Further support of the utility of engaging in exercise training at a consistent time-of-day is observed through our findings related to changes in self-reported fatigue and quality of life. In the total sample we observed that engaging in habitual AM or PM exercise was associated with significant improvements in fatigue and quality of life. There was insufficient evidence to observe an association between those engaging in a mix of AM and PM exercise and quality of life or fatigue. While not statistically significant in breast cancer survivors for these variables, changes in self-reported fatigue among survivors who habitually engaged in AM and PM exercise were greater compared to those engaging in a mix of exercise training time. Furthermore, changes in quality of life among breast cancer survivors who habitually engaged in AM and PM exercise were the same and greater compared with mixed exercise training.

Engagement in a mix of exercise training time throughout the program was associated with a significant increase in waist circumference, and among breast cancer survivors, a significant increase in body weight and BMI. Weight gain is of particular importance for breast cancer survivors, as weight gain after breast cancer diagnosis is associated with poor prognosis [26]. Preclinical evidence and human observational data support the relationship between circadian rhythm disruption and incidence of obesity and metabolic disease [41]. Thus, these findings support the notion that an inconsistent, or mixed, exercise training time among cancer survivors may result in less attenuation of circadian rhythm disruption secondary to cancer treatment.

Waist circumference is a proxy measure for visceral adiposity, and excessive accumulation of visceral adiposity is associated with poor prognosis and survival outcomes across varying cancer types, including breast cancer [42–45]. Among the total sample, while not statistically significant, greater reductions in waist circumference were observed with AM exercise training compared with PM. These findings are consistent with literature among individuals with obesity [21, 22]. In contrast, while not statistically significant, among breast cancer survivors, greater reductions in waist circumference were observed with PM versus AM training. It is possible that the habitual time-of-day of exercise training, AM or PM, in the context of weight management may be different in cancer survivors, depending on cancer type, compared with other clinical populations. More work should be done to confirm these findings and determine mechanisms that may underpin the differences for cancer survivors compared with individuals with other chronic diseases such as obesity.

A strength of this study includes exploring the association between time-of-day of exercise training and changes in relevant cancer health outcomes among cancer survivors specifically (as this is the first study, to our knowledge, to assess this relationship in the context of cancer). Additional strengths include measuring timing data rather than self-report, and objective measurement of health outcomes. Limitations of the present investigation include heterogeneity of the sample, the unbalanced sample size for each variable assessed, retrospective nature of the analysis, limited data related to change in other cancer health outcomes such as glycemic control, and the fact that the majority of exercise completed during training sessions in the program is resistance training since this is where guidance is most needed. Participants are coached on aerobic exercise, but participants primarily complete this independently, and the program does not document the time of day of this exercise. Additionally, we must acknowledge that these results provide an initial assessment of habitual exercise timing within an exercise program and changes in outcomes measured at the beginning and end of the program in cancer survivors. It is possible that the observed findings are due to the exercise timing since it is known that exercise can manipulate these outcomes in cancer survivors [1]; however, no adjustments were made for multiple comparisons since this is an initial assessment. Therefore, we cannot infer causality from this retrospective analysis. Further, MCID assessment included thresholds established in other clinical populations and not cancer survivors, as MCID thresholds in cancer survivors have yet to be established. Future work should consider a larger, more homogenous sample and a prospective study design. There are varying mechanisms that may explain the observed associations; however, more research is needed to identify clear-cut associations and underlying mechanisms.

## Conclusions

Overall, we observed that time-of-day of exercise training might differentially impact human performance and physical function among cancer survivors. In comparison, consistent engagement in morning or afternoon/evening exercise training may improve self-reported fatigue and quality of life. In contrast, inconsistent exercise training time, or mixed exercise training time, may result in less favorable outcomes of weight management variables among breast cancer survivors specifically.

## Supporting information

S1 File Minimal anonymized Data_Coletta et al exercise timing paper. (XLSX)
